# Supplementary material for: Silver and Gold Complexes with NHC-Ligands Derived from Caffeine: Catalytic and Pharmacological Activity
Source: Int J Mol Sci. 2024 Feb 23;25(5):2599. doi: 10.3390/ijms25052599 (PMC10931745; doi:10.3390/ijms25052599)
Supplement: Supplementary file 1 [file ijms-25-02599-s001.zip › ijms-2885951-SI.pdf]

Supporting Information

For

Silver and Gold Complexes with NHC-Ligands Derived from Caffeine: Catalytic and Pharmacological Activity

Annaluisa Mariconda [1], Domenico Iacopetta [2], Marco Sirignano [3], Jessica Ceramella [2], Assunta D'Amato [3], Maria Marra [2], Michele Pellegrino [2], Maria Stefania Sinicropi [2,*], Stefano Aquaro [2,†] and Pasquale Longo [3,†]

[1] Department of Science University of Basilicata Viale dell'Ateneo Lucano 10, 85100 Potenza, Italy

[2] Department of Pharmacy, Health and Nutritional Sciences University of Calabria, Via Pietro Bucci, 87036 Arcavacata di Rende, Italy

[3] Department of Chemistry and Biology "A. Zambelli" University of Salerno, Via Giovanni Paolo II 132, 84084 Fisciano, Italy

† These authors contributed equally to this work.

* Correspondence: s.sinicropi@unical.it

List of contents

^{1}H, ^{13}C – NMR and mass spectra..4

9-[(2-hydroxy-2-phenyl)ethyl]-1,3,7-trimethylxanthinium iodide (P-L1) ... 4
^{1}H-NMR ... 4
ESI-MS ... 5
9-[cyclohexan-2-ol]-1,3,7-trimethylxantinium iodide (P-L2) ... 7
^{1}H-NMR ... 7
^{13}C-NMR ... 8
MALDI-MS ... 8
9-[(2-hydroxy)ethyl)]-1,3,7-trimethylxantinium iodide (P-L3) ... 9
^{1}H-NMR ... 9
^{13}C-NMR ... 10
MALDI-MS ... 10
1,3,7-trimethylxanthin-9-[(2-hydroxy-2-phenyl)ethyl-8-ylidene]Ag(I) acetate (AgL1OAc) ... 12
^{1}H-NMR ... 12
^{13}C-NMR ... 13
MALDI-MS ... 13
1,3,7-trimethylxanthin-9-[cyclohexan-2-ol-8-ylidene]Ag(I) acetate (AgL2OAc) ... 14
^{1}H-NMR ... 14
^{13}C-NMR ... 14
MALDI-MS ... 15
1,3,7-trimethylxanthin-9-[(2-hydroxy)ethyl)-8-ylidene]Ag(I) acetate (AgL3OAc) ... 16
^{1}H-NMR ... 16
^{13}C-NMR ... 17
MALDI-MS ... 17
1,3,7-trimethylxanthin-9-[(2-hydroxy-2-phenyl)ethyl-8-ylidene]Au(I) iodide (AuL1) ... 18
^{1}H-NMR ... 18
^{13}C-NMR ... 19
MALDI-MS ... 19
1,3,7-trimethylxanthin-9-[cyclohexan-2-ol-8-ylidene]Au(I) iodide (AuL2) ... 20
^{1}H-NMR ... 20
^{13}C-NMR ... 21
MALDI-MS ... 21
1,3,7-trimethylxanthin-9-[(2-hydroxy)ethyl)-8-ylidene]Au(I) iodide (AuL3) ... 22
^{1}H-NMR ... 22
^{13}C-NMR ... 22
MALDI-MS ... 24
1,3,7-trimethylxanthin-9-[(2-hydroxy-2-phenyl)ethyl-8-ylidene]Au(I) acetate (AuL1OAc) ... 25

^{1}H-NMR 25
^{13}C-NMR 26
MALDI-MS 26
***Proligands Bioactivity* 27**

^{1}H, ^{13}C – NMR and mass spectra

9-[(2-hydroxy-2-phenyl)ethyl]-1,3,7-trimethylxanthinium iodide (P-L1)

^{1}H-NMR

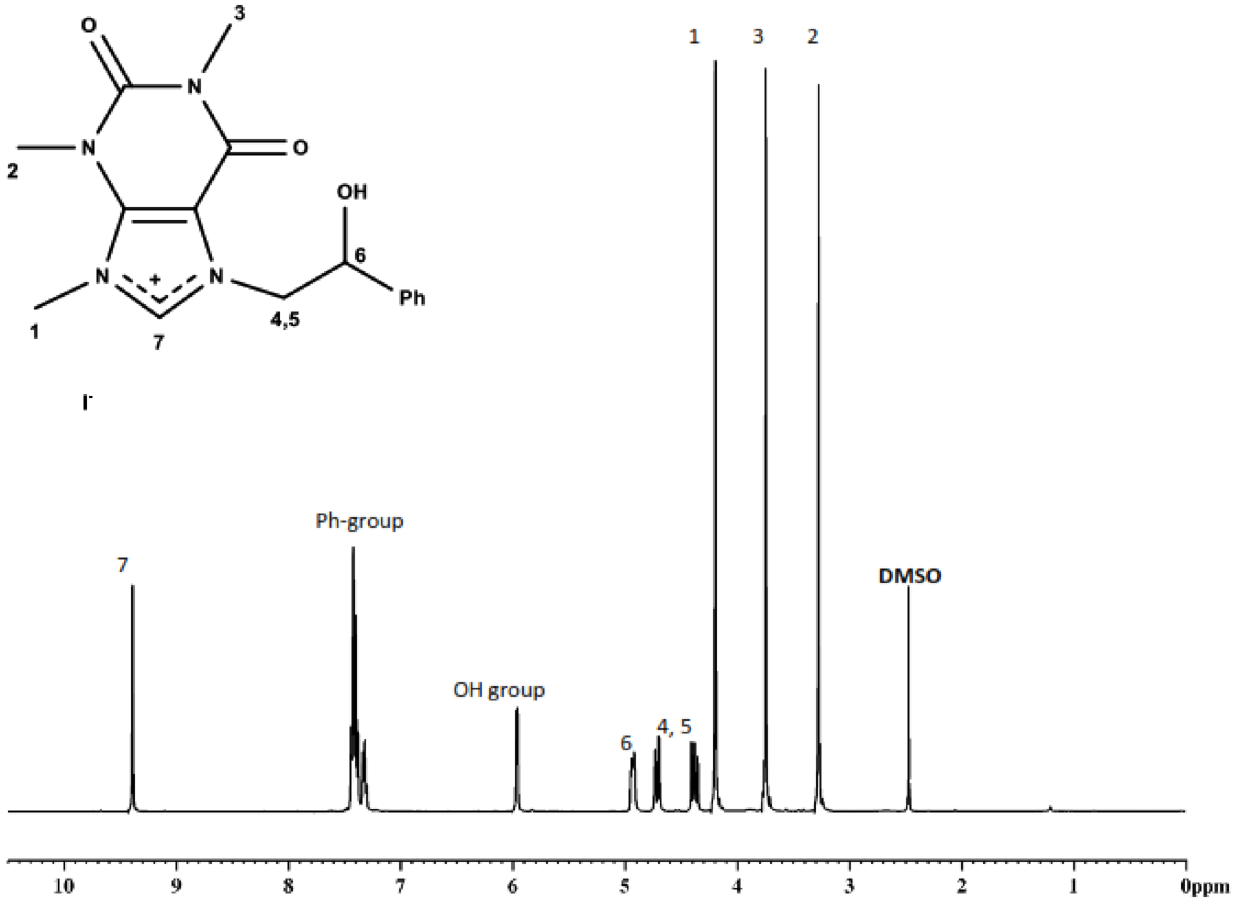

^{1}H-NMR (400 MHz, DMSO-d$_6$): δ 9.39 (s, 1H, NC***H***N), 7.44-7.30 (m, 5H, *Ph-group*), 5.96 (s, 1H, O***H***), 4.73 (s, 1H, C***H***OH), 4.69-4.40 (m, 2H, NC***H₂***), 4.35 (s, 3H, NC***H₃*** imidazolium ring), 3.74 (s, 3H, NC***H₃***), 3.27 (s, 3H, NC***H₃***).

^{13}C-NMR

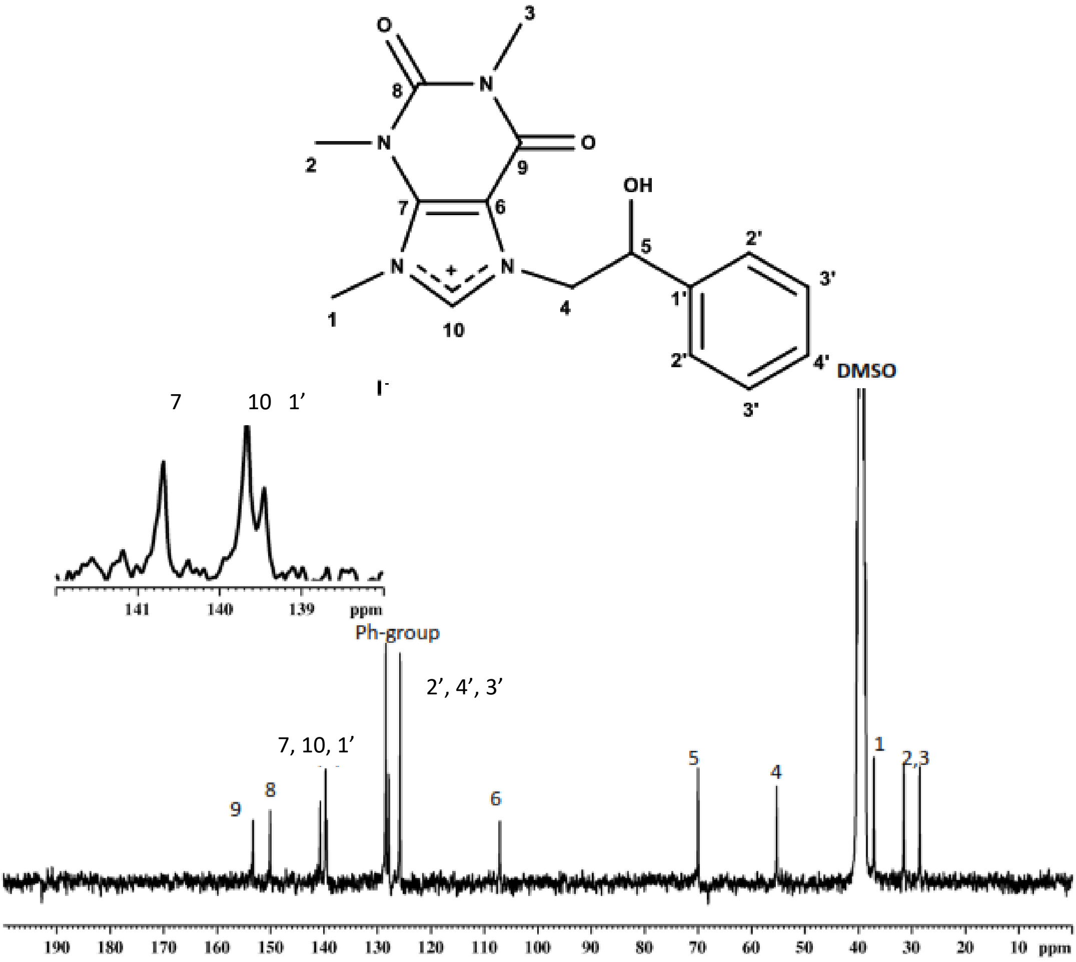

^{13}C-NMR (100 MHz, DMSO-d_6): δ 153.3_3 and 150.1_9 (***C***=O *purine ring*), 140.9_3 (*backbone carbon*, CH_3N***C***=C) 139.9_3 (N***C***HN), 139.5_5 *(ipso aromatic carbon*, **Ph-ring**), 128.2_6 (*aromatic carbons*, **Ph ring**), 128.2_1 (*aromatic carbon*, **Ph ring**), 125.8_7 (*aromatic carbons*, **Ph ring**), 108.8_2 (*backbone carbon*, C=***C***NCH_2), 70.3_0 (***C***HOH), 55.4_5 (N***C***H_2), 36.9_3 (N***C***H_3 imidazolium *ring*) 31.4_8 and 28.3_6 (N***C***H_3 purine ring).

ESI-MS

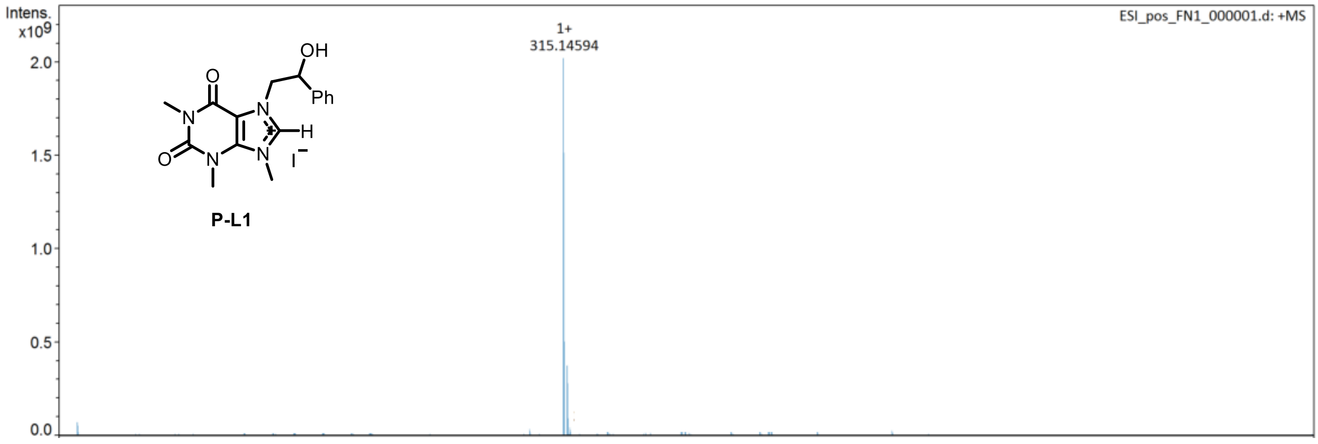

ESI-MS (CH_3CN m/z) **=** 315.14594 attributable to the cationic part of imidazolium proligand $[C_{16}H_{19}N_4O_3]^+$.

9-[cyclohexan-2-ol]-1,3,7-trimethylxantinium iodide (P-L2)

^{1}H-NMR

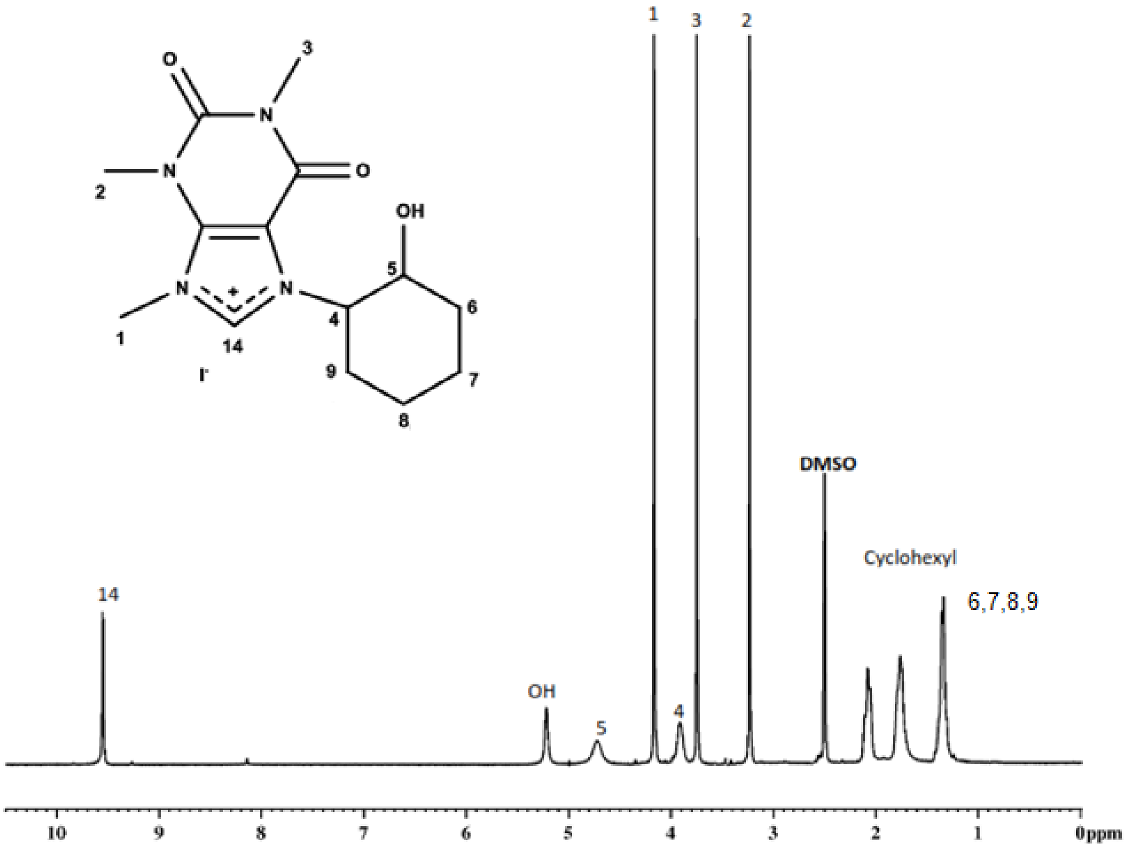

^{1}H-NMR (400 MHz, DMSO-d$_6$): δ 9.54 (s, 1H, NC***H***N), 5.26 (s, 1H, O***H***), 4.78-4.70 (br, 1H, C***H***OH), 4.16 (s, 3H, NC***H₃*** imidazolium ring), 3.91-3.90 (br, 1H, NC***H*)*, 3.75 (s, 3H, NC***H₃***), 3.28 (s, 3H, NC***H₃***), 2.10-175 (m, 8H, *Cyclohexyl*).

^{13}C-NMR

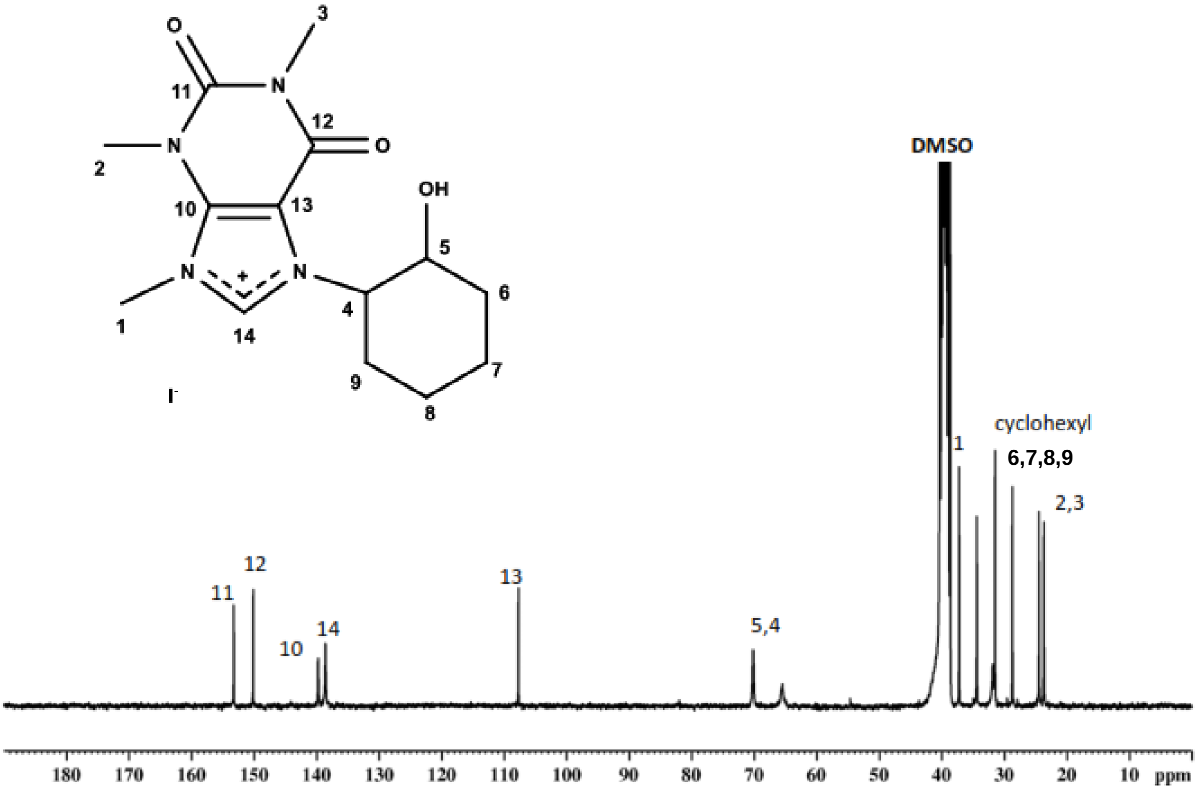

^{13}C-NMR (100 MHz, DMSO-d$_6$): *δ* 153.2_0-150.1_2 (***C***=O), 139.6_9 (*backbone carbons*, CH$_3$N***C***=C), 138.5_5 (N***C***HN), 107.7_0 (*backbone carbons*, C=***C***NCH$_2$), 70.1_6 (***C***HOH), 65.4_7 (N***C***H), 37.2_4 (N***C***H$_3$ imidazolium *ring*), 34.4_1-28.7_2, (*cyclohexyl carbons*), 24.4_4 (N***C***H$_3$ purine ring), 23.6_7 (N***C***H$_3$ purine ring).

MALDI-MS

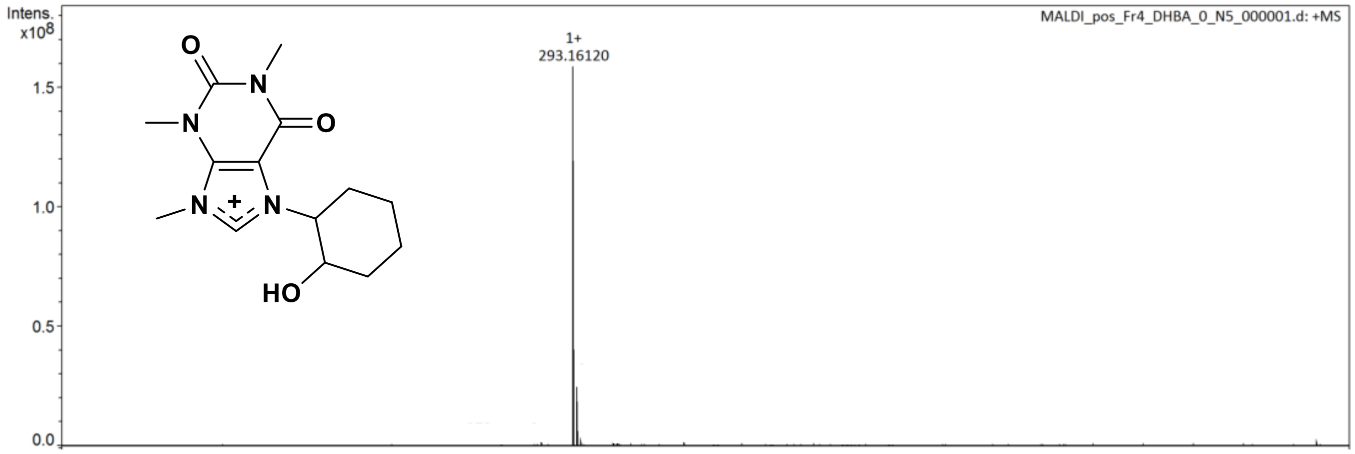

MALDI-ToF(m/z): 293.16137 Da attributable to the cationic part of imidazolium proligand $[C_{14}H_{21}N_4O_3]^+$

9-[(2-hydroxy)ethyl)]-1,3,7-trimethylxantinium iodide (P-L3)

^{1}H-NMR

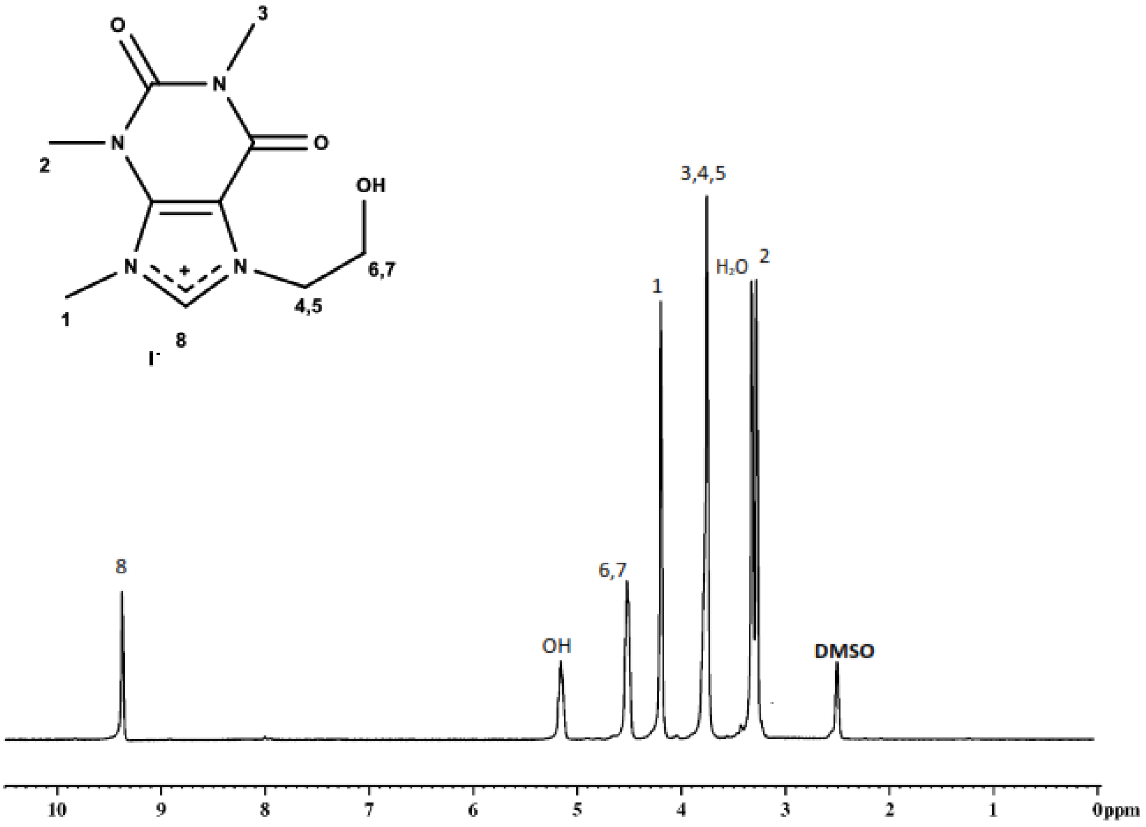

^{1}H-NMR (400 MHz, DMSO-d_6)**:** δ 9.37 (s, 1H, NC***H***N), 5.16 (m, 1H, O***H***), 4.52-4.50 (m, 2H, C***H₂***OH), 4.19 (s, 3H, NC***H₃*** imidazolium ring), 3.75 (m, 5H, NC***H₂,*** N***C***H_3), 3.27 (s, 3H, NC***H₃***).

^{13}C-NMR

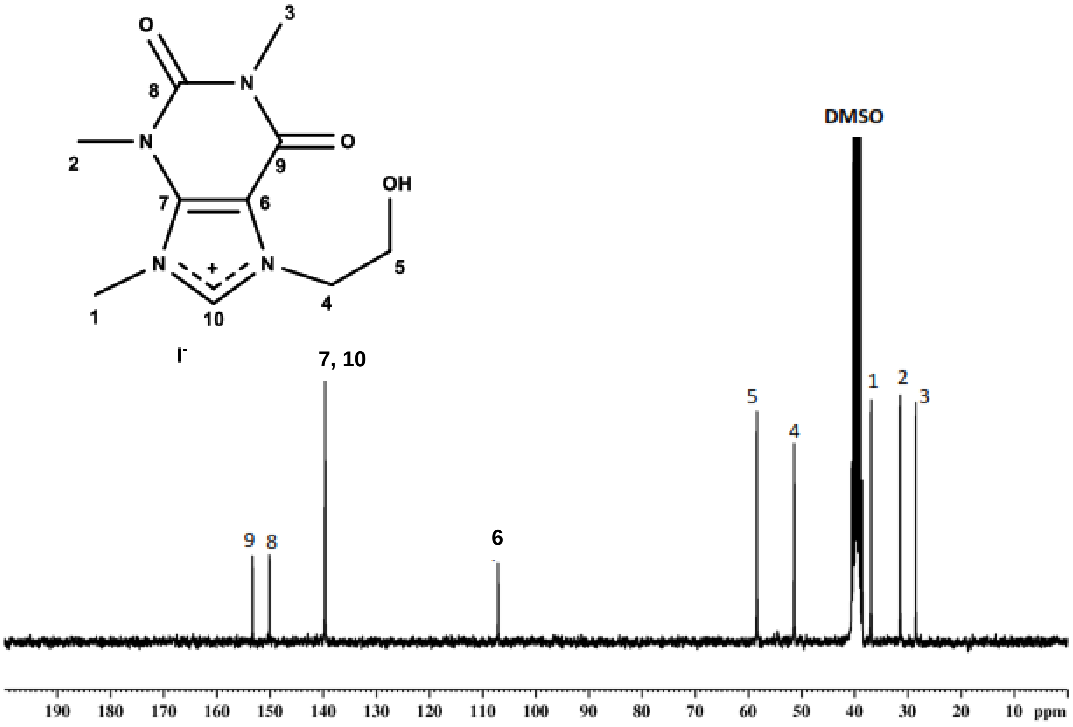

^{13}C-NMR (75 MHz, DMSO-d_6): δ 153.2_3 and 150.1_2 (***C***=O), 139.6_3 (*backbone carbon*, CH_3N***C***=C and N***C***HN), 107.0_3 (*backbone carbon*, *C*=***C****N*), 58.3_5 (***C***H_2OH), 51.3_9 (N***C***H_2), 36.9_1 (N***C***H_3 imidazolium *ring*), 31.4_0 (N***C***H_3 purine ring), 28.4_9 (N***C***H_3 purine ring).

MALDI-MS

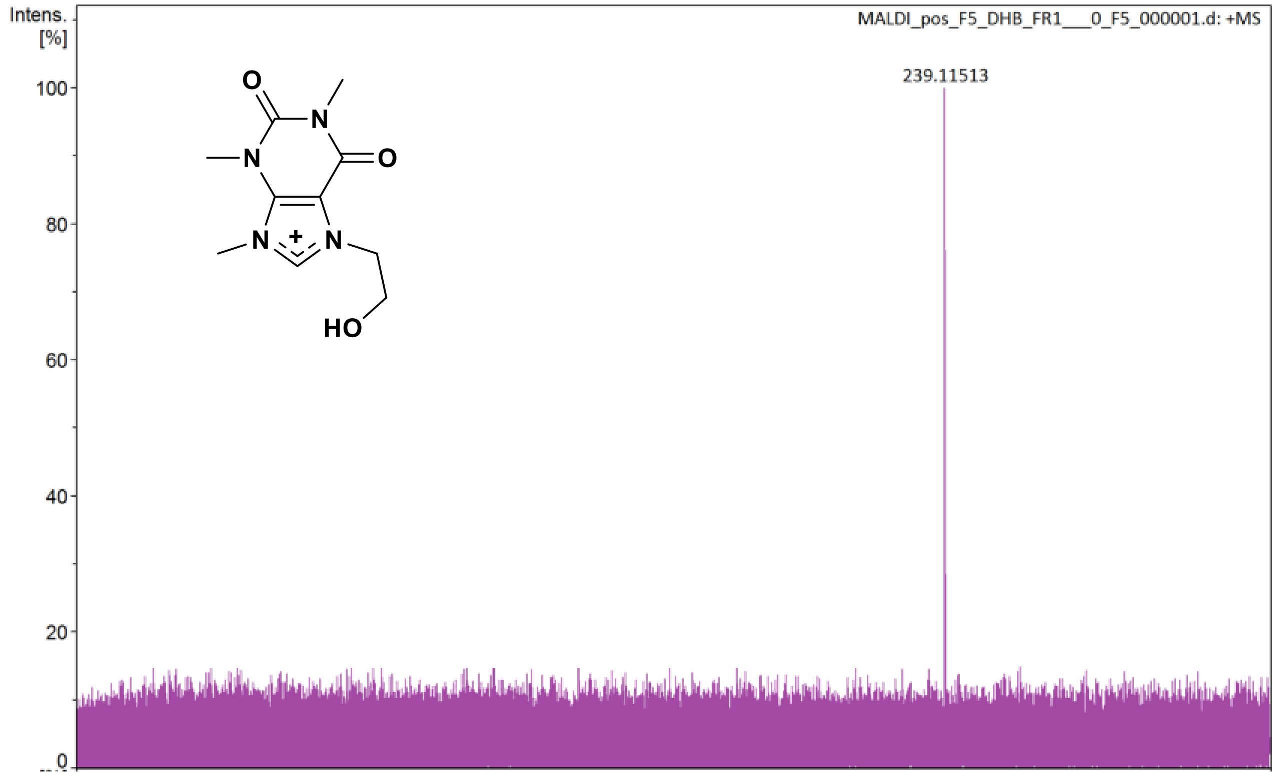

MALDI-ToF (m/z): 239.11513 Da attributable to cationic part of imidazolium proligand $[C_{10}H_{15}N_4O_3]^+$

1,3,7-trimethylxanthin-9-[(2-hydroxy-2-phenyl)ethyl-8-ylidene]Ag(I) acetate (AgL1OAc)

^{1}H-NMR

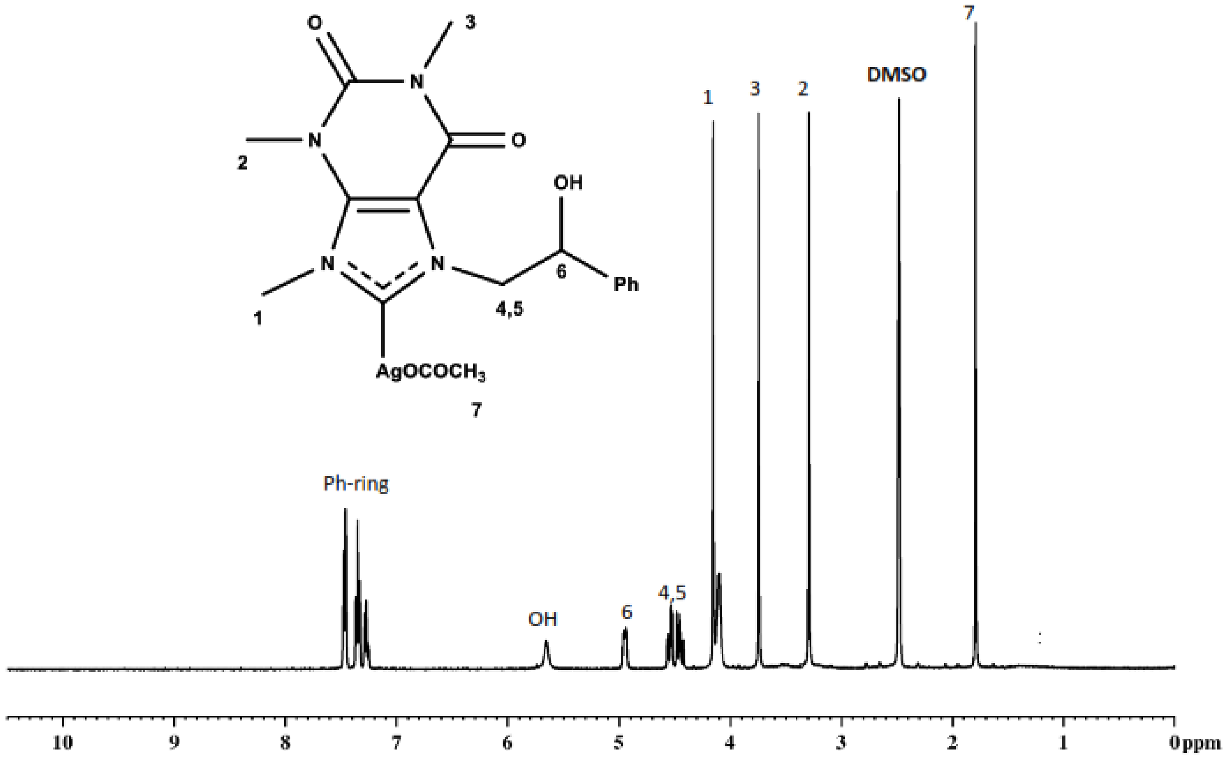

^{1}H-NMR (400 MHz, DMSO-d_6)**:** δ 7.41-7.29 (m, 5H, ***Ph-group***), 5.62 (s, 1H, O***H***), 4.93-4.89 (m, 1H, C***H***OH), 4.60-4.32 (m, 2H, NC***H***$_2$), 4.19 (s, 3H, NC***H***$_3$ imidazole carbene), 3.75 (s, 3H, NC***H***$_3$), 3.27 (s, 3H, NC***H***$_3$), 1.77 (s, 3H, O=CC***H***$_3$).

^{13}C-NMR

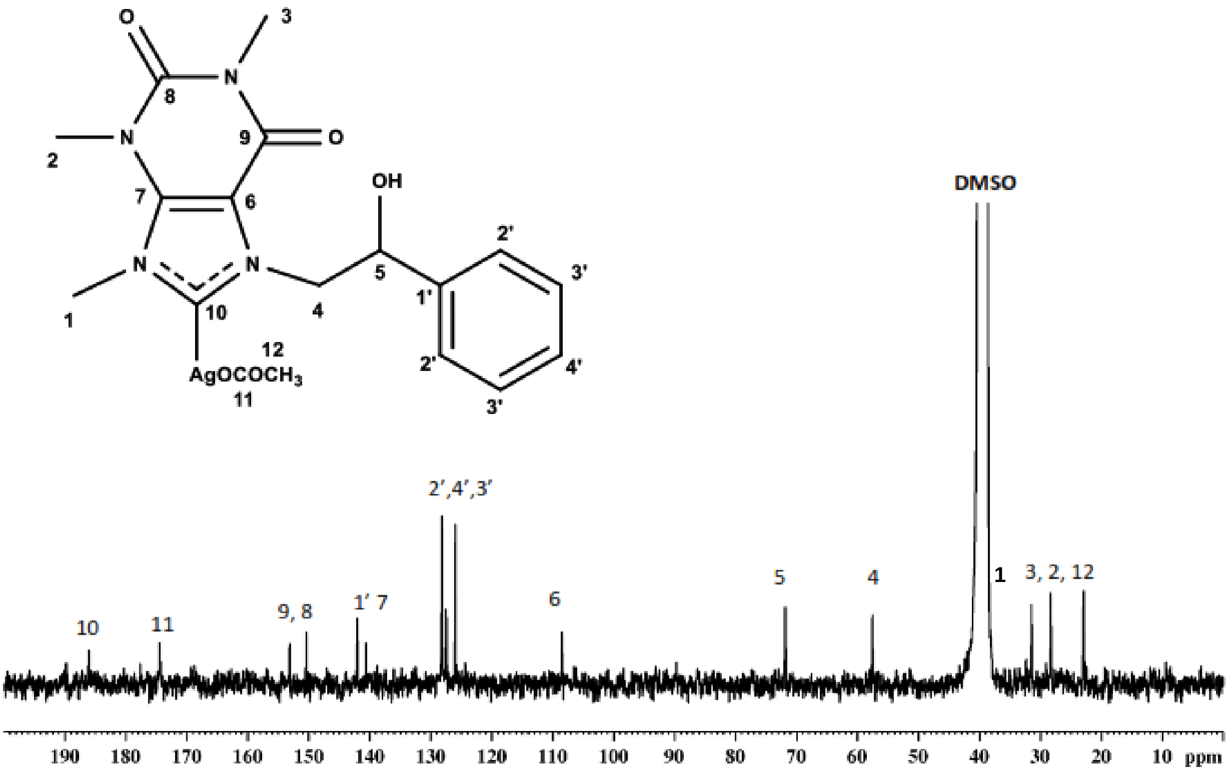

^{13}C-NMR (100 MHz, DMSO-d$_6$): δ 186.3$_6$ (N**C**N), 174.5$_1$ (CH$_3$**C**=O), 153.3$_3$-150.3$_9$ (**C**=O *purine ring*), 142.4$_5$ *(ipso aromatic carbon*, **Ph-ring**), 140.6$_3$ (*backbone carbon*, CH$_3$N**C**=C), 128.2$_6$, 128.1$_1$, 125.7$_7$ (*aromatic carbons*, **Ph ring**), 108.8$_2$ (*backbone carbon*, C=**C**NCH$_2$), 72.1$_0$ (**C**HOH), 57.1$_5$ (N**C**H$_2$), 37.3$_7$ (N**C**H$_3$), 31.4$_8$ and 28.3$_6$ (N**C**H$_3$ *purine ring*), 22.9$_6$ (O=C**C**H$_3$).

MALDI-MS

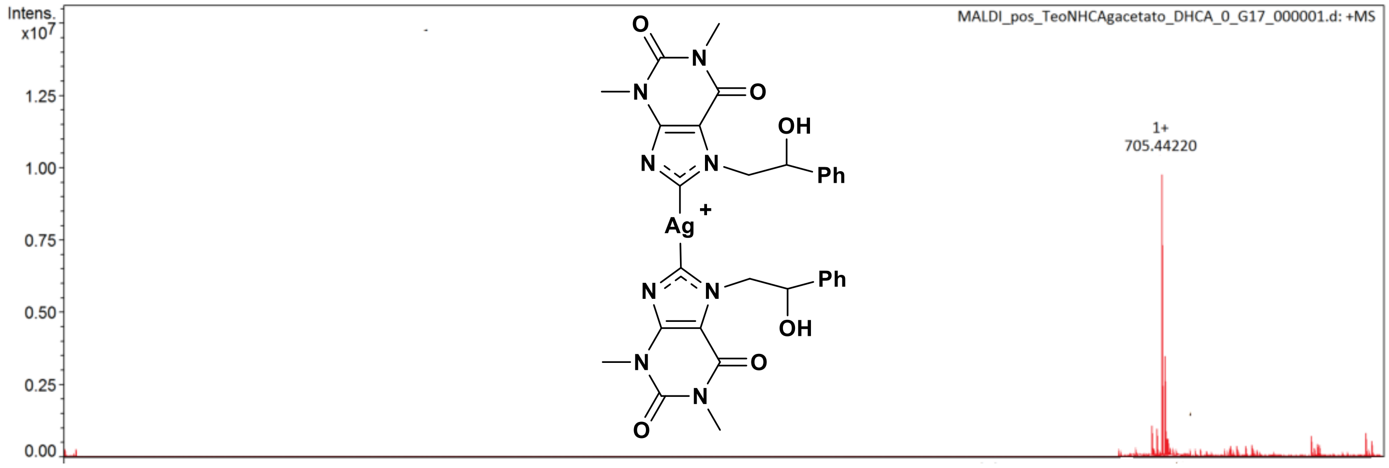

MALDI-ToF (m/z): 705.44220 Da attributable to silver bis-carbenic structure $[C_{30}H_{30}AgN_8O_6]^+$

1,3,7-trimethylxanthin-9-[cyclohexan-2-ol-8-ylidene]Ag(I) acetate (AgL2OAc)

^{1}H-NMR

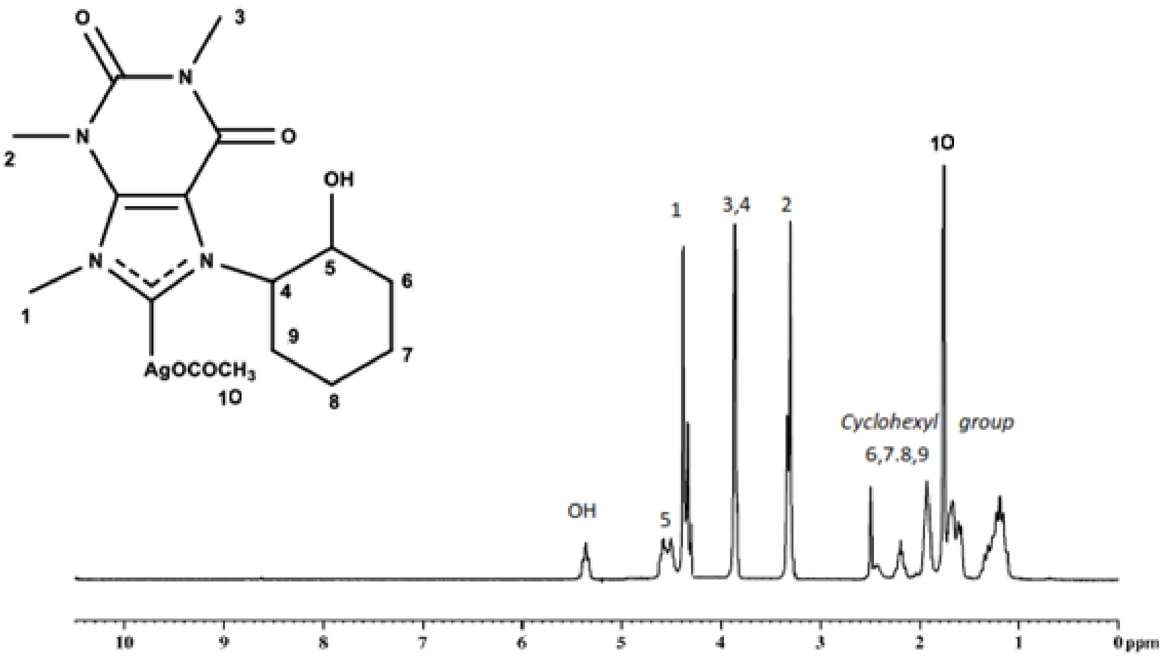

^{1}H-NMR (400 MHz, DMSO-d$_6$)**:** δ 5.36 (m, 1H, O***H***), 4.63-4.55 (m, 1H, C***H***OH), 4.36 (s, 3H, NC***H$_3$*** imidazole carbene), 3.86 (o, 4H, NC***H***, N***CH$_3$***), 3.25 (s, 3H, NC***H$_3$***), 2.10-1.75 (m, 11H, *Cyclohexyl* + O=CC***H$_3$***).

^{13}C-NMR

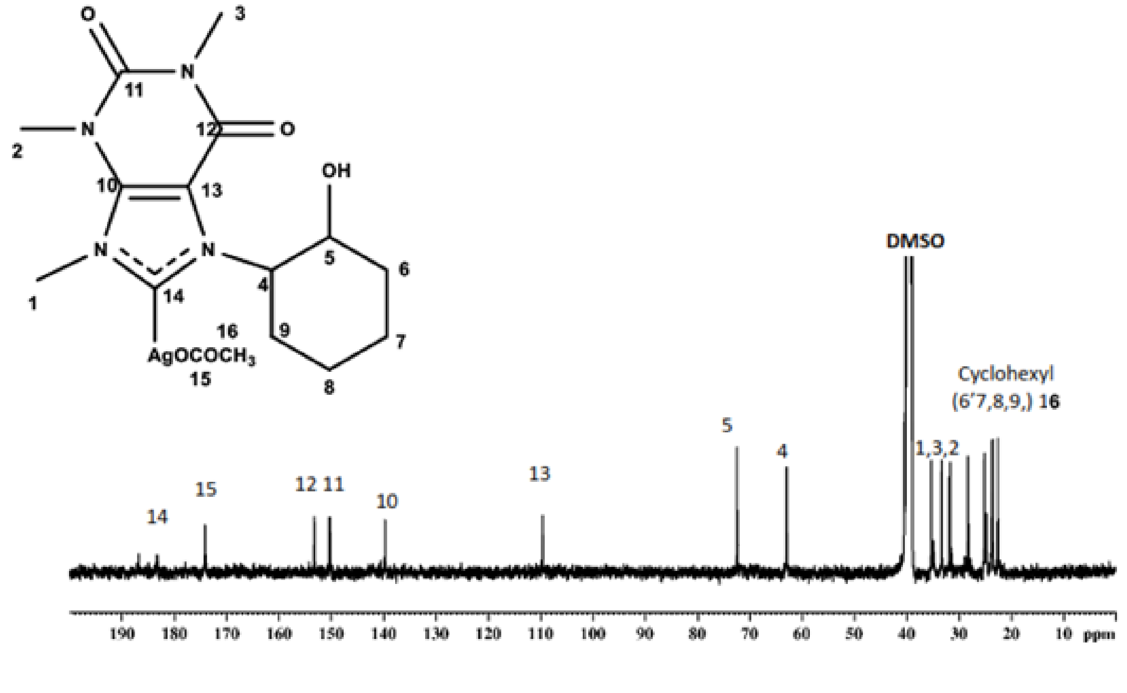

^{13}C-NMR (100 MHz, DMSO-d$_6$): *δ* 183.8$_5$ (N**C**N), 175.4$_0$ (O=***C***CH$_3$), 153.2$_3$ and 150.2$_2$ (***C***=O *purine ring*), 139.6$_7$ (*backbone carbon* CH$_3$N***C***=C), 109.5$_7$ (*backbone carbon* C=***C***NCH$_2$), 72.3$_3$ (O**C**H), 62.9$_0$ (N**C**H), 37.8$_0$ (N***C***H$_3$), 33.4$_0$ and 28.4$_9$ (N***C***H$_3$ *purine ring*), 34.0$_2$-23.5$_5$ (*Cyclohexyl group*), 22.8$_2$ (O=C***C***H$_3$).

MALDI-MS

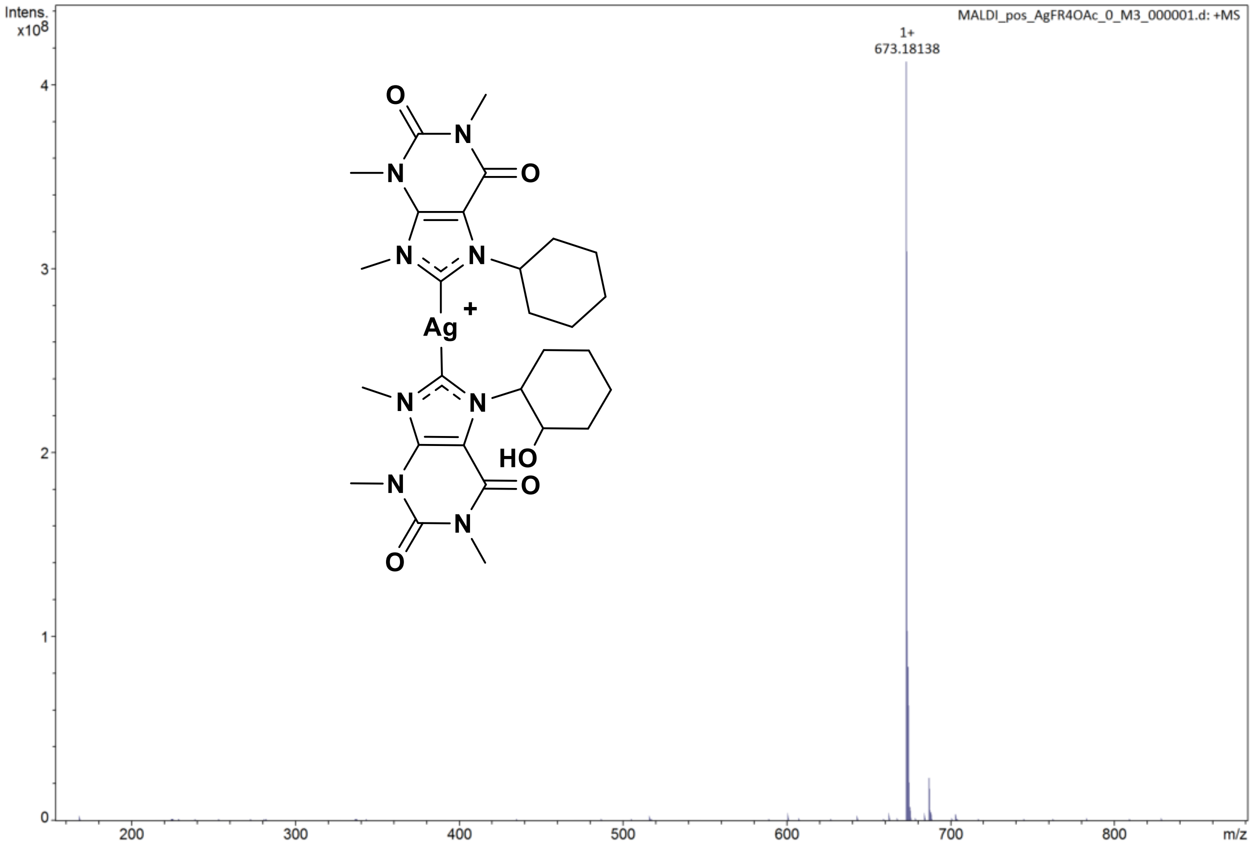

MALDI-ToF (m/z): 673.18138 Da attributable to silver bis-carbenic structure $[C_{28}H_{39}AgN_8O_6]^+$ (Ag(NHC)$_2$ -OH)

1,3,7-trimethylxanthin-9-[(2-hydroxy)ethyl)-8-ylidene]Ag(I) acetate (AgL3OAc)

^{1}H-NMR

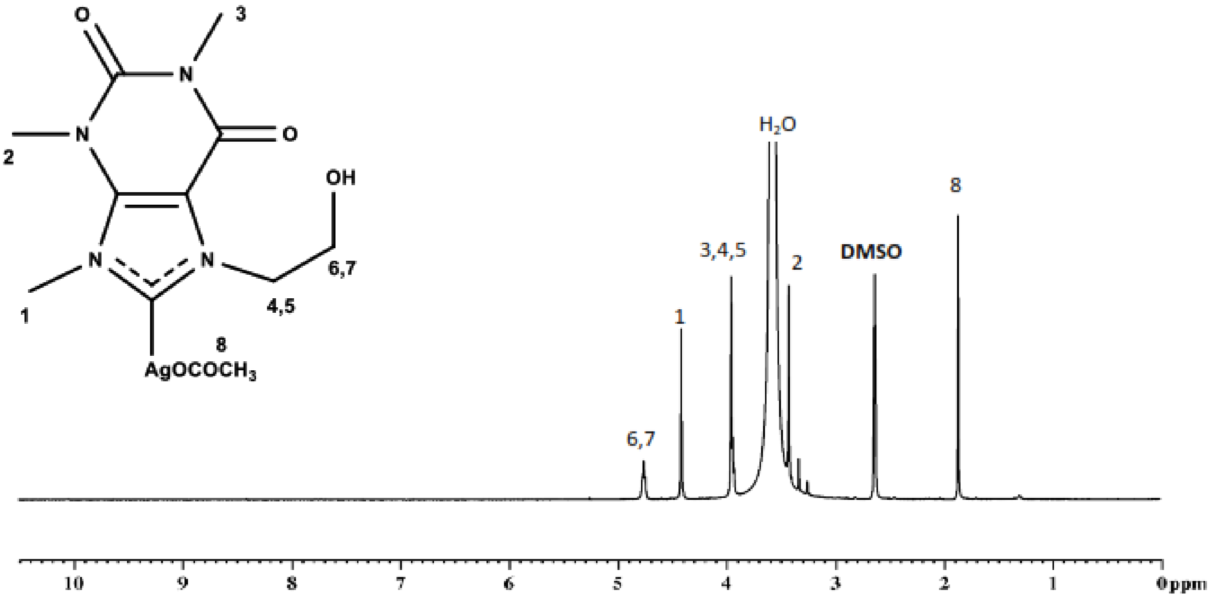

^{1}H-NMR (400 MHz, DMSO-d$_6$)**:** δ 4.73-4.60 (m, 2H, C***H₂***OH), 4.49 (s, 3H, NC***H₃*** imidazole carbene), 3.96 (o, 5H, NC***H₂,*** NC***H₃***), 3.28 (s, 3H, NC***H₃***), 1.87 (s, 3H, O=CC***H₃***).

^{13}C-NMR

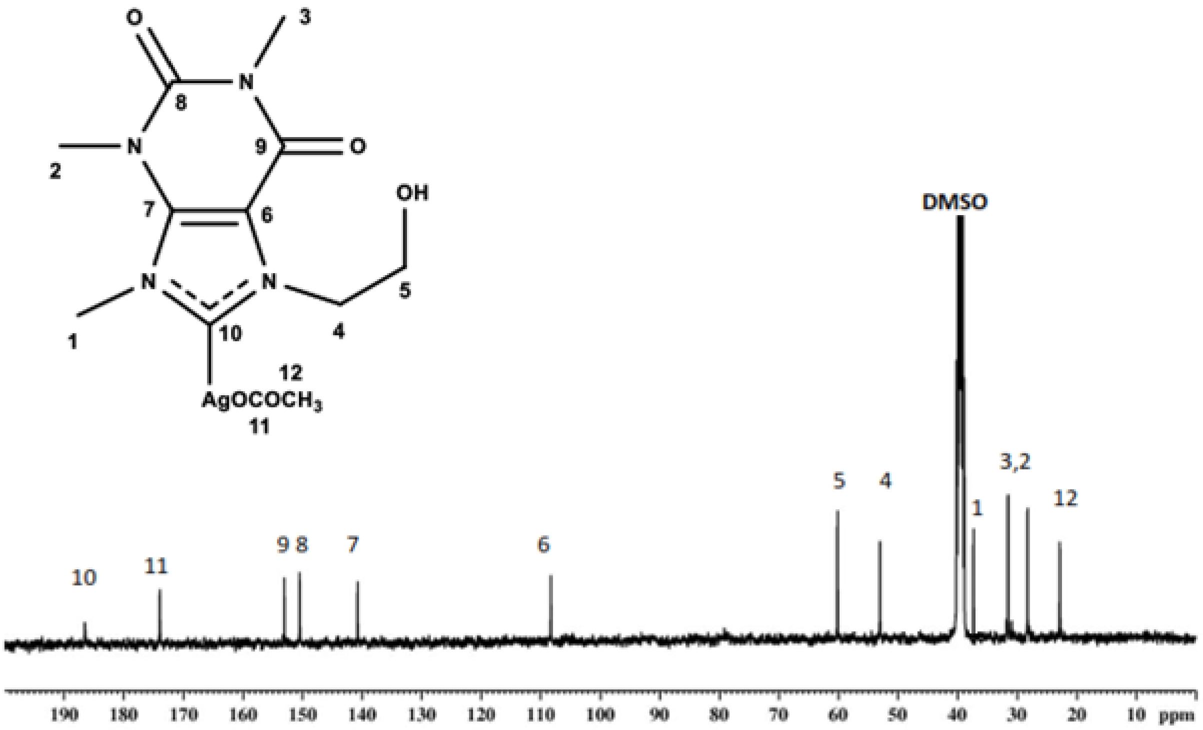

^{13}C-NMR (400 MHz, DMSO-d_6)**:** δ 186.5_8 (N**C**N); 175.9_0 (O=**C**CH_3); 153.0_3 and150.4_2 (**C**=O *purine ring*); 140.7_2 (*backbone carbon* CH_3N**C**=C); 108.2_3 (*backbone carbon*, C=**C**NCH_2) 60.1_2 (**C**H_2OH), 52.9_9 (N**C**H_2), 37.69 (N**C**H_3, imidazole carbene), 31.4_0 and 28.4_9 (N**C**H_3 *purine ring*), 22.8_2 (O=C**C**H_3).

MALDI-MS

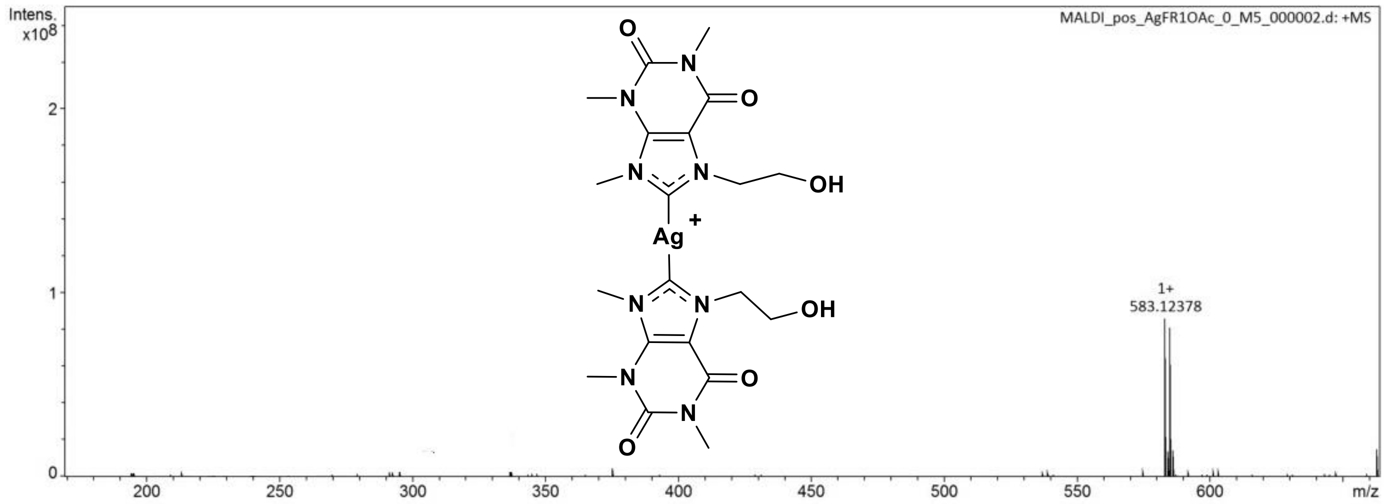

MALDI-ToF (m/z): 583.12378 Da attributable to silver bis-carbenic structure $[C_{20}H_{28}AgN_8O_6]^+$

1,3,7-trimethylxanthin-9-[(2-hydroxy-2-phenyl)ethyl-8-ylidene]Au(I) iodide (AuL1)

^{1}H-NMR

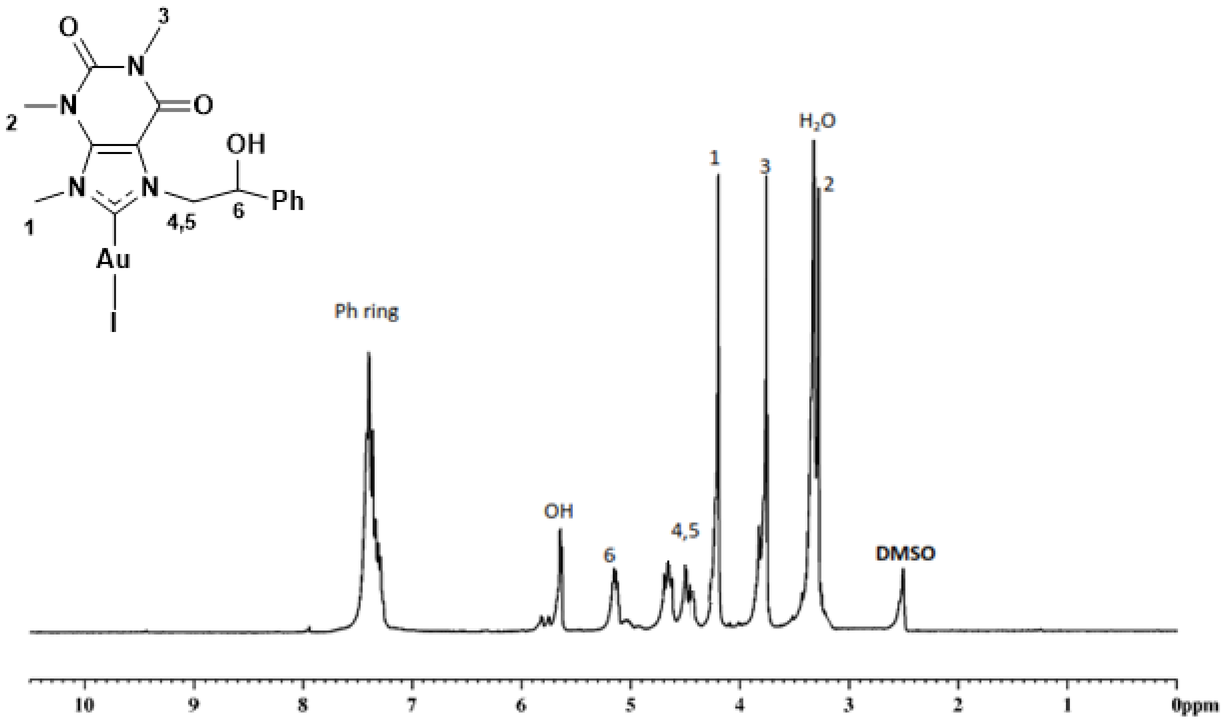

^{1}H-NMR (400 MHz, DMSO-d$_6$): δ 7.41-7.29 (m, 5H, ***Ph-group***), 5.62 (s, 1H, O***H***), 5.13 (m, 1H, C***H***OH), 4.60-4.32 (m, 2H, NC***H₂***), 4.19 (s, 3H, NC***H₃*** imidazole carbene), 3.75 (s, 3H, NC***H***$_3$), 3.27 (s, 3H, NC***H***$_3$).

^{13}C-NMR

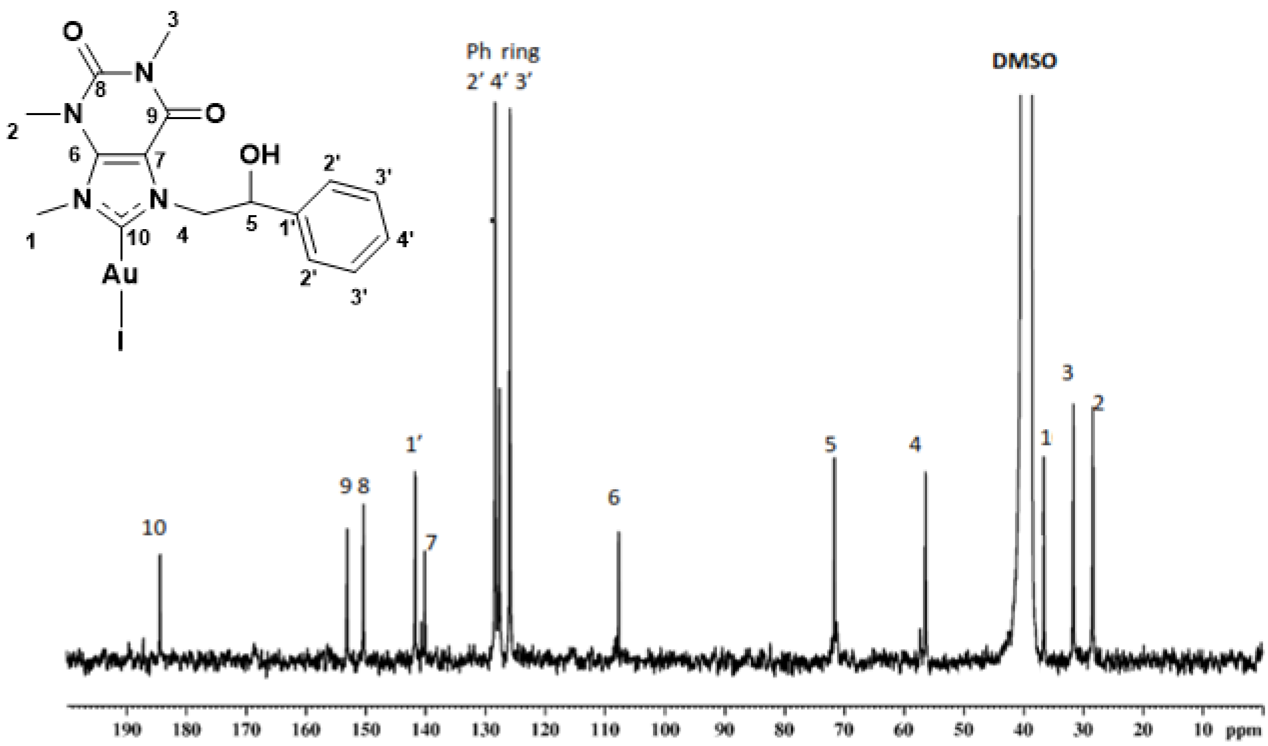

^{13}C-NMR (400 MHz, DMSO-d$_6$): δ 184.0_0 (N***C***N), 153.5_3 and 150.4_9 (***C***=O purine ring), 141.7_5 (ipso aromatic carbon, ***Ph-ring***), 139.8_3 (backbone carbon, C=***C***NCH$_2$), 128.3_6, 127.6_1, 125.8_7 (aromatic carbons, ***Ph ring***), 107.6_2 (backbone carbon, CH$_3$N***C***=C), 71.6_0 (***C***HOH), 56.3_5 (N***C***H$_2$), 38.2_1 (N***C***H$_3$ imidazole carbene), 31.6_8 and 28.3_6 (NC***H$_3$*** purine ring).

MALDI-MS

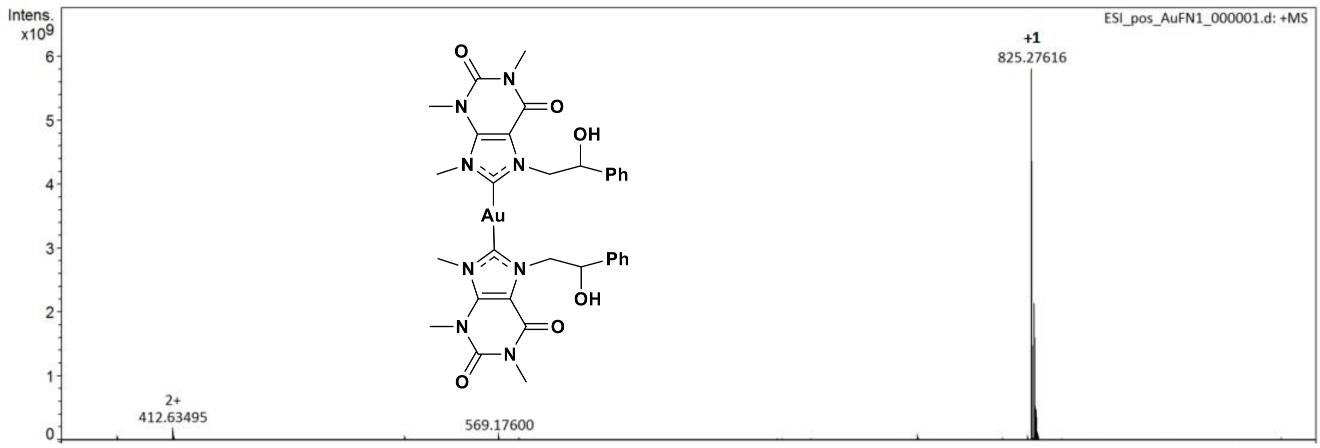

MALDI-MS (m/z): 825.27616 Da attributable to a bis-carbenic gold complex structure [$C_{32}H_{26}AuN_8O_6$]$^+$

1,3,7-trimethylxanthin-9-[cyclohexan-2-ol-8-ylidene]Au(I) iodide (AuL2)

^{1}H-NMR

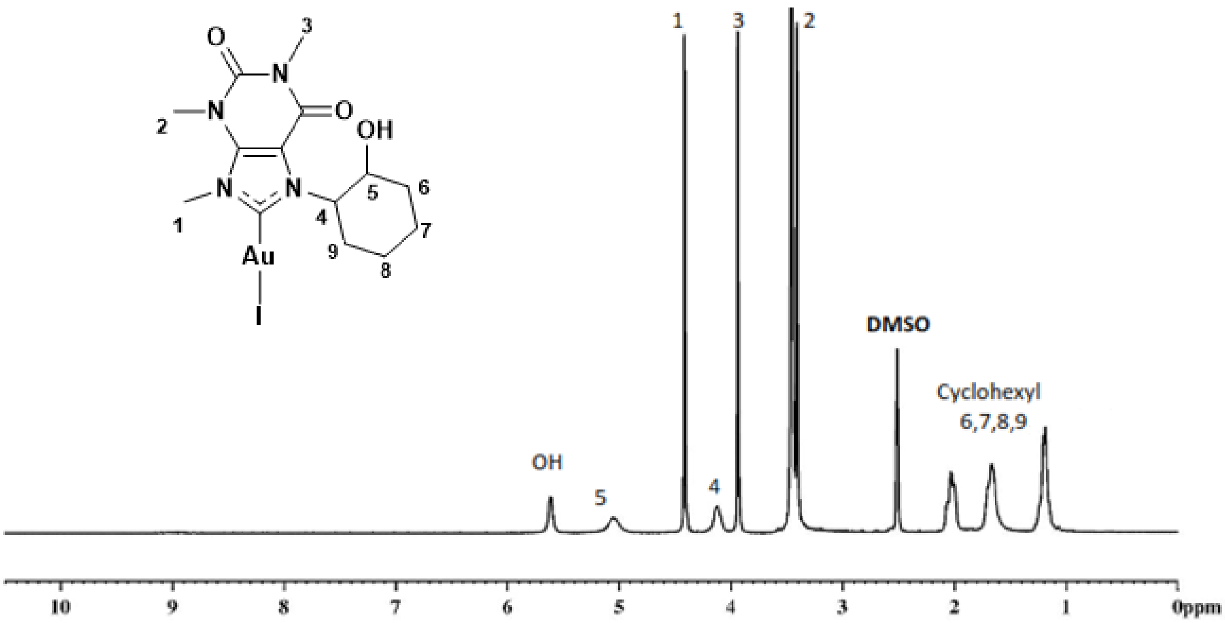

^{1}H-NMR (400 MHz, DMSO-d$_6$)**:** δ 5.56 (s, 1H, O***H***), 5.17 (b, 1H, C***H***OH), 4.26 (s, 3H, NC***H*₃** imidazole carbene), 4.08 (b, 1H, NC***H***), 3.86 (s, 3H, NC***H*₃**), 3.25 (s, 3H, NC***H*₃**), 2.20-1.75 (m, 11H, ***Cyclohexyl***).

^{13}C-NMR

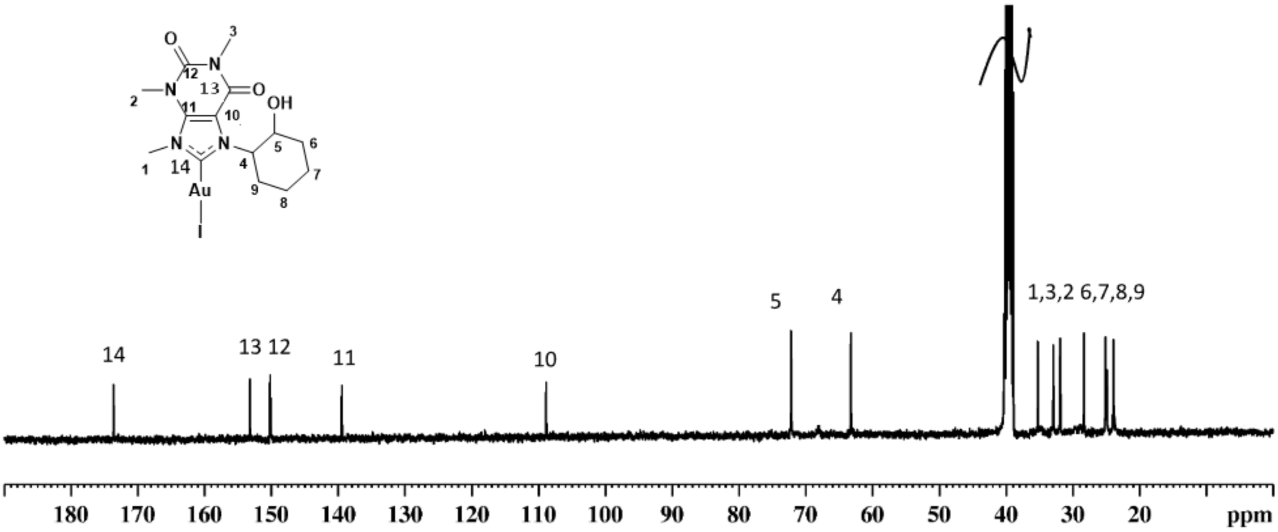

^{13}C-NMR (100 MHz, DMSO-d$_6$): δ 175.6$_5$ (N***C***N), 153.3$_3$ and 149.2$_2$ (***C***=O *purine ring*), 139.6$_7$ (*backbone carbon CH$_3$N**C**=C*), 108.7$_7$ (*backbone carbon* C=***C***NCH), 72.3$_3$ (O***C***H), 62.9$_0$ (N***C***H), 36.8$_0$ (N***C***H$_3$ imidazole carbene), 33.4$_0$ and 32.6$_5$ (N***C***H$_3$ *purine ring*), 28.4$_9$, 25.4$_2$, 25.3$_7$, 23.5$_5$ (*Cyclohexyl group*).

MALDI-MS

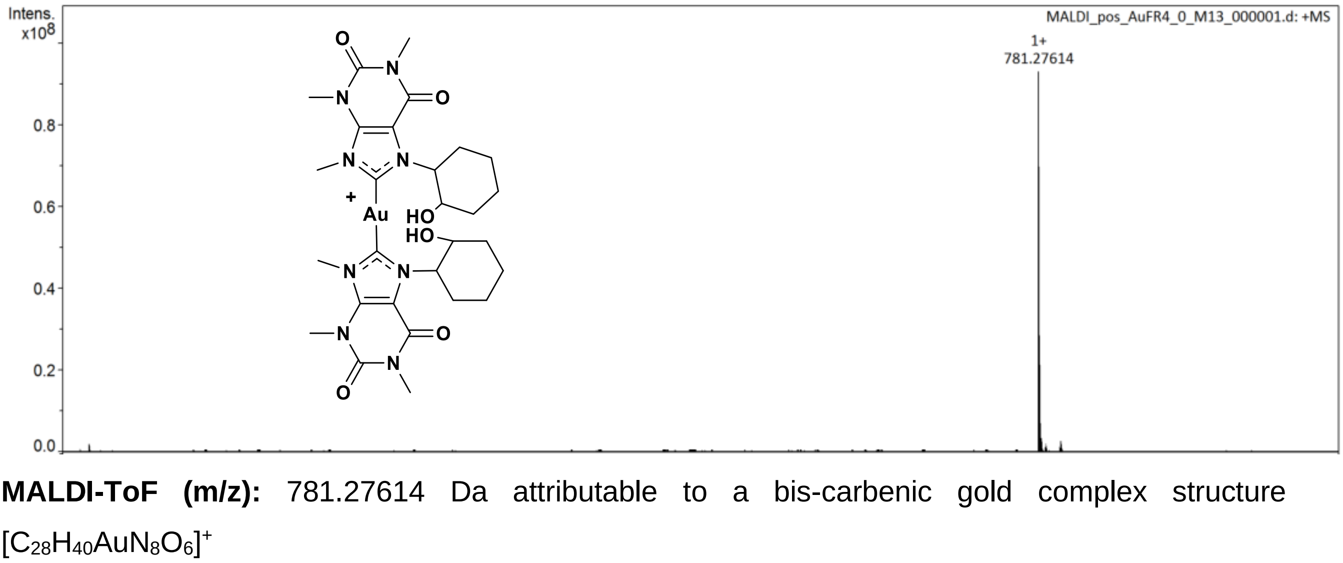

MALDI-ToF (m/z): 781.27614 Da attributable to a bis-carbenic gold complex structure [$C_{28}H_{40}AuN_8O_6$]$^+$

1,3,7-trimethylxanthin-9-[(2-hydroxy)ethyl)-8-ylidene]Au(I) iodide (AuL3)

^{1}H-NMR

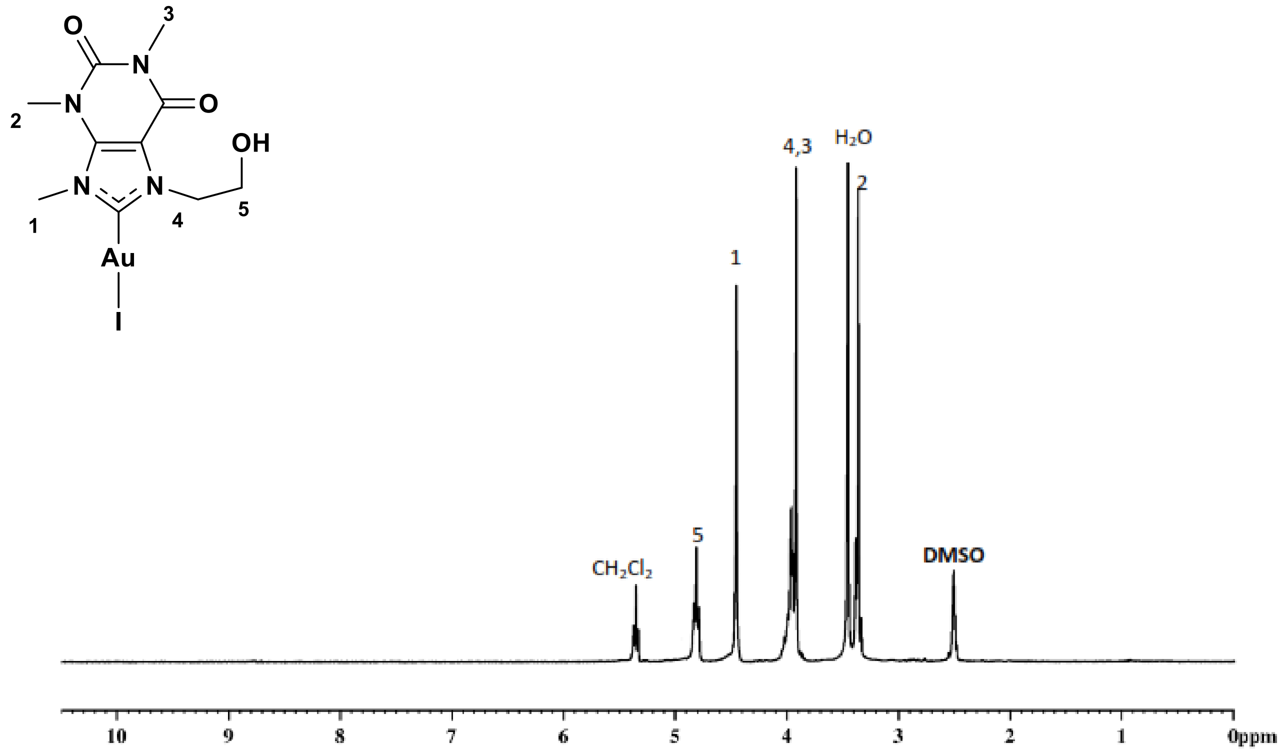

^{1}H-NMR (400 MHz, DMSO-d$_6$)**:** δ 4.83-4.76 (m, 2H, C***H*₂**OH), 4.49 (s, 3H, NC***H*₃** imidazole carbene), 3.86-3.73 (o, 5H, NC***H*₂,** NC***H*₃**), 3.26 (s, 3H, NC***H*₃**).

^{13}C-NMR

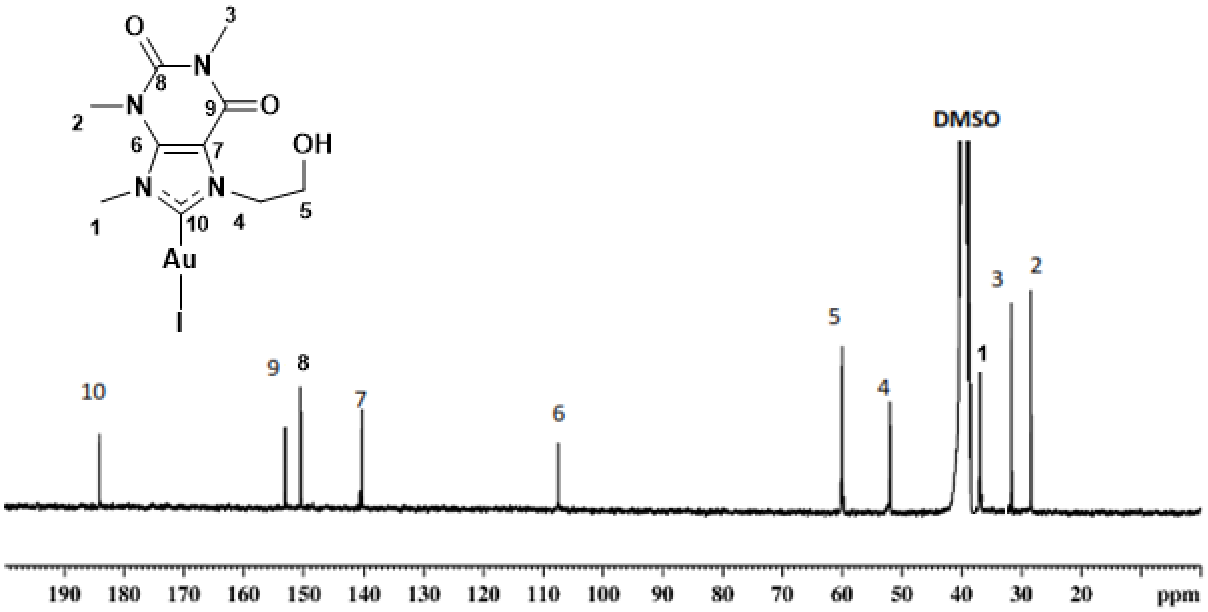

^{13}C-NMR (100 MHz, DMSO-d_6)**:** *δ* 184.6_8 (N***C***N); 153.4_3 and 150.3_2 (***C***=O *purine ring*); 139.7_2 (*backbone carbon* CH_3N***C***=C); 107.2_3 (*backbone carbon*, C=***C***NCH_2), 60.2_2 (***C***H_2OH), 52.9_9 (N***C***H_2), 39.4_5 (N***C***H_3), 31.5_1, 28.2_4 (N***C***H_3 imidazole carbene), 31.4_0 and 28.4_9 (N***C***H_3 *purine ring*).

MALDI-MS

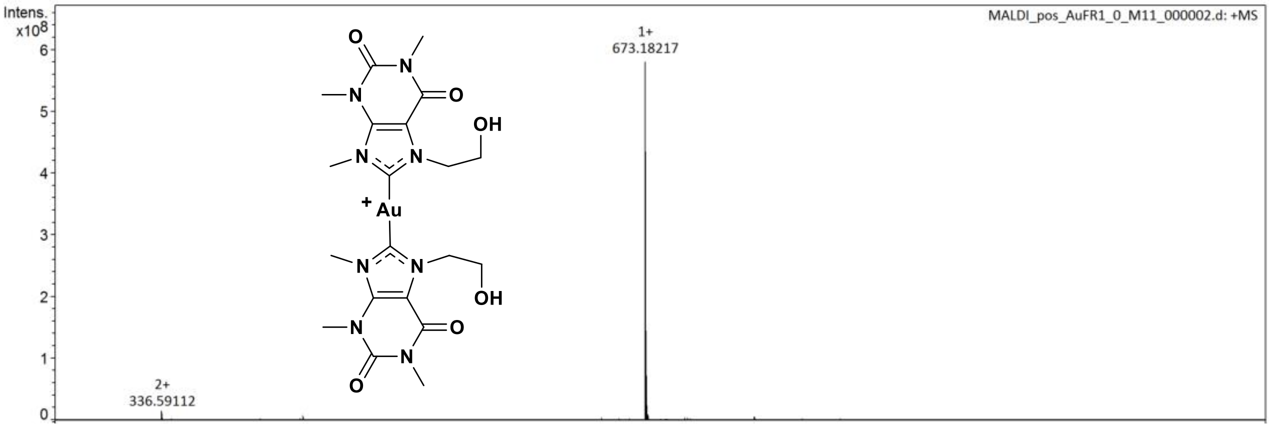

MALDI-ToF (m/z): 673.18217 Da attributable to a bis-carbenic gold complex structure $[C_{20}H_{28}AuN_8O_6]^+$

1,3,7-trimethylxanthin-9-[(2-hydroxy-2-phenyl)ethyl-8-ylidene]Au(I) acetate (AuL1OAc)

^{1}H-NMR

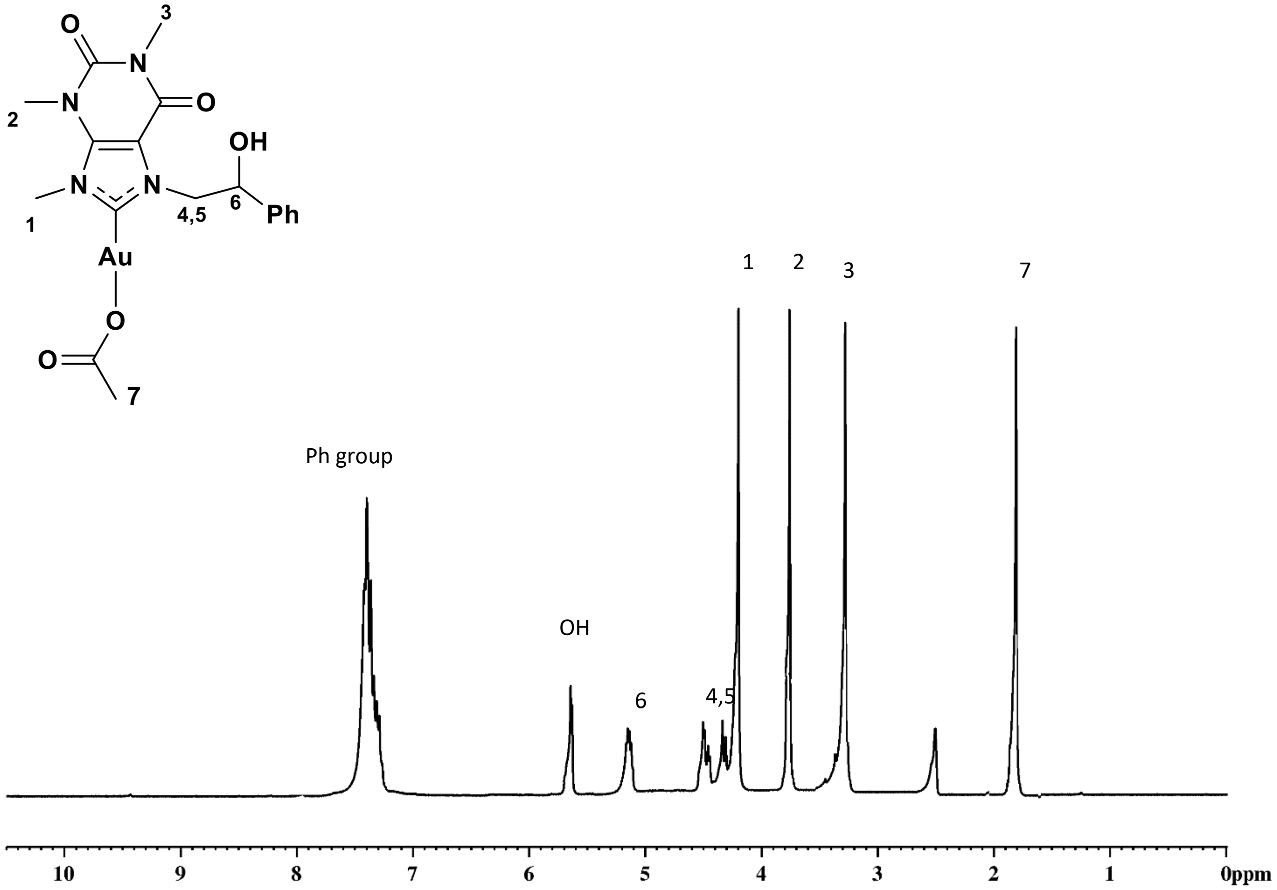

^{1}H-NMR (400 MHz, DMSO-d_6)**:** *δ* 7.39-7.28 (m, 5H, ***Ph-group***), 5.62 (s, 1H, O***H***), 5.13 (m, 1H, C***H***OH), 4.60-4.32 (m, 2H, NC***H***$_2$), 4.19 (s, 3H, NC***H***$_3$ imidazole carbene), 3.75 (s, 3H, NC***H***$_3$), 3.27 (s, 3H, NC***H***$_3$), 1.79 (s, 3H, O=CC***H***$_3$).

^{13}C-NMR

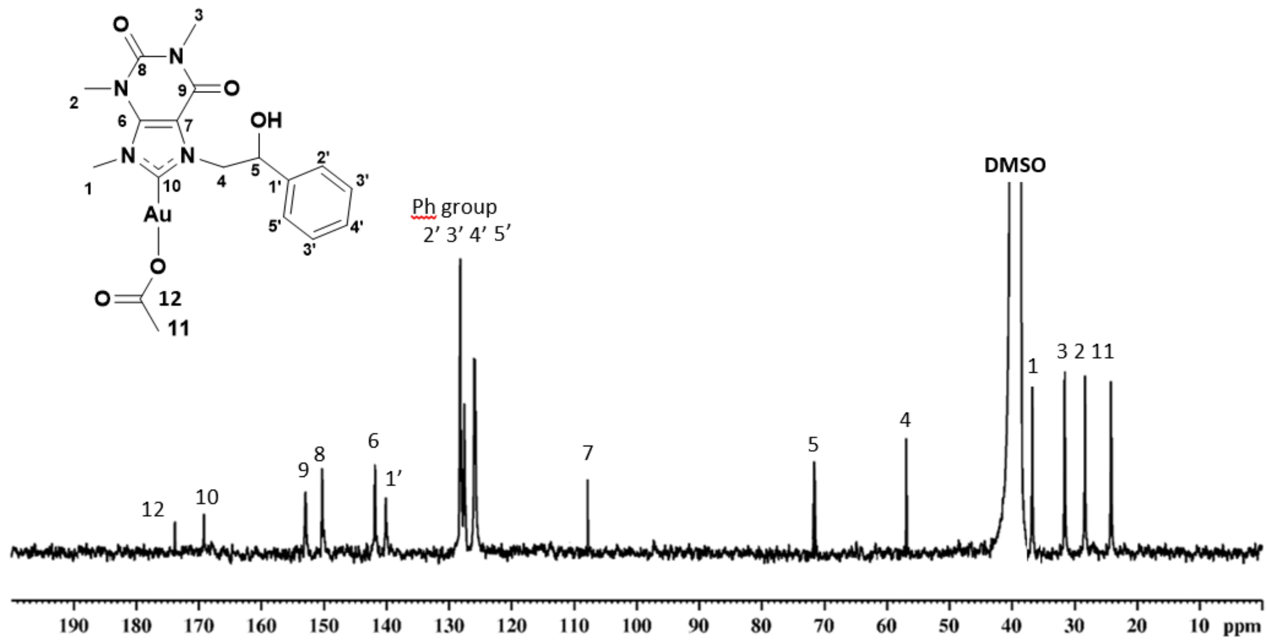

^{13}C-NMR (100 MHz, DMSO-d$_6$): δ 173.9_0 (O=***C***CH$_3$), 168.3_8 (N***C***N), 153.3_3 and 149.2_2 (***C***=O purine ring), 142.1_7 (*backbone carbon*, CH$_3$N***C***=C) 140.0_7 *(ipso aromatic carbon*, **Ph-ring**), 131.9_7-125.5_9 (*aromatic carbons*, **Ph ring**), (*backbone carbon*, C=***C***NCH$_2$) 72.1_6 (O***C***H), 56.0_2 (N***C***H$_2$), 39.4_2 (N***C***H$_3$ imidazole carbene), 37.3_7 and 28.3_6 (N***C***H$_3$ purine ring), 22.8_1 (O=C***C***H$_3$).

MALDI-MS

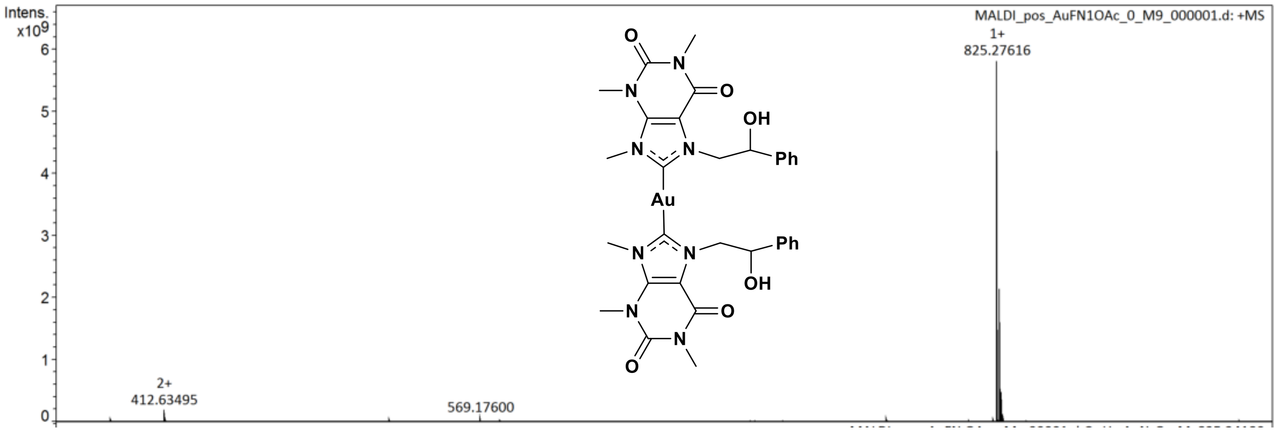

MALDI-ToF (m/z): 825.27616 attributable to a bis-carbenic gold complex structure [$C_{32}H_{26}AuN_8O_6$]$^+$

Proligands Bioactivity

IC$_{50}$ (µM)			
	MDA-MB-231	MCF-7	MCF-10A
**P-L1**	>100	>100	>100
**P-L2**	>100	>100	>100
**P-L3**	>100	>100	>100

Table S1. IC$_{50}$ values, expressed in µM, for **P-L1-3** on three different cell lines (MCF-7, MDA-MB-231, MCF-10A). The values represent the mean± standard deviation of three different experiments, performed in triplicate.

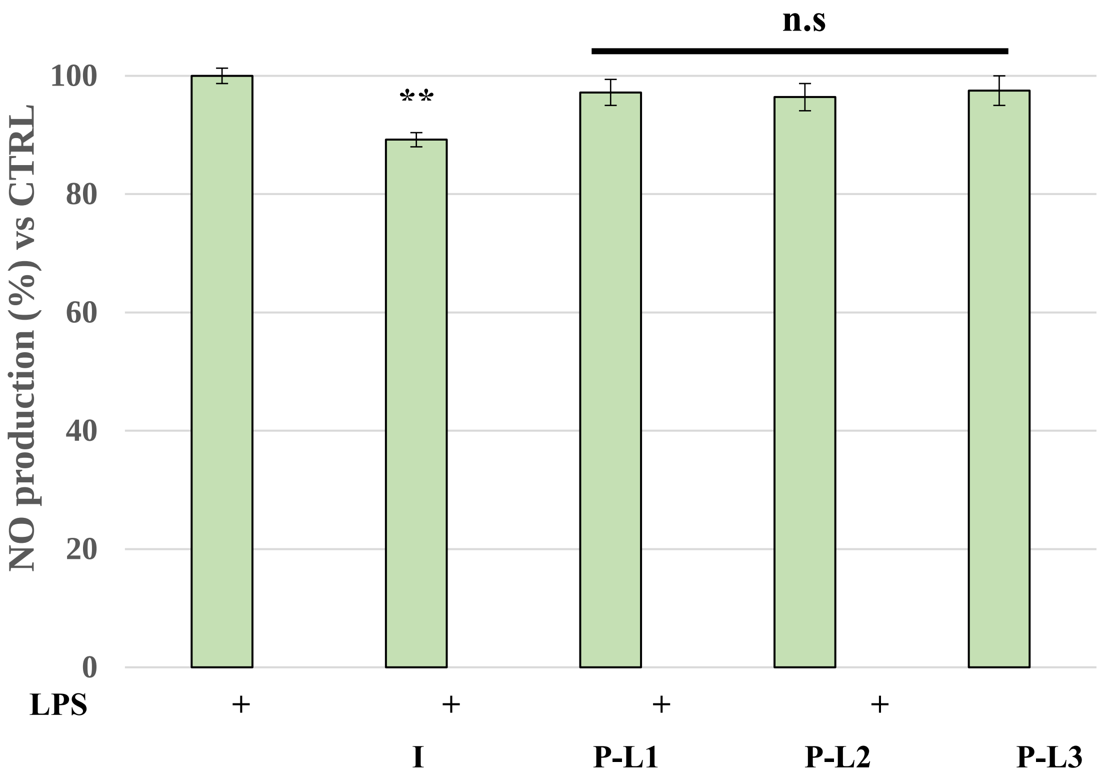

Figure S1. Anti-inflammatory activity in terms of NO production (%) measured *in vitro* using the murine macrophages RAW 264.7. NO production was induced using LPS (1 µg/mL). The proligands **P-L1-3** and indomethacin (I) were used at the concentration of 25 µM. **I** and **P-L1-3** *vs* LPS, **p<0.005, n.s. (not significant).

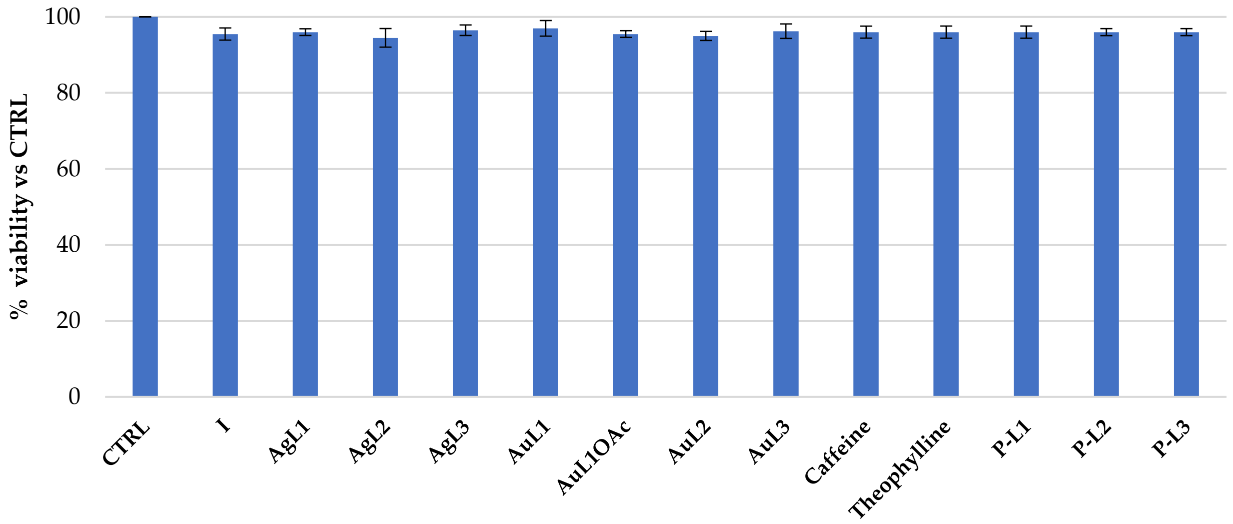

Figure S2. Viability assay (MTT). Murine macrophages RAW 264.7 viability determination after 24 h of treatment (%, treated *vs* CTRL), repeated trice. The complexes **Ag(L1-3)OAc** and **Au(L1-3)**, caffeine, theophylline, indomethacin (I) and proligands **P-L1-3** were used at the concentration of 25 µM. n.s. (not significant), treated *vs* CTRL.
